# Validity of Rapid Antibody Testing for COVID-19 Vaccine in Homeless People

**DOI:** 10.3390/v15061400

**Published:** 2023-06-20

**Authors:** Se-Min Hwang, Yun Jung, Jiyeon Seo, Yoomi Jung, Shinae Park, Haesook Seo

**Affiliations:** 1 Department of Preventive Medicine, Konyang University College of Medicine, Daejeon 35365, Republic of Korea; neofreud2@daum.net; 2 Graduate School of Public Health & Welfare, Konyang University, Daejeon 35365, Republic of Korea; 3 Myunggok Medical Research Center, Konyang University College of Medicine, Daejeon 35365, Republic of Korea; 4 Health Promotion Division, Guro Public Health Center, Seoul 08299, Republic of Korea; rubyyun68@guro.go.kr; 5 Health Examination Center, Seoul Metropolitan Government, Seobuk Hospital, Seoul 03433, Republic of Korea; pobbii4@seoul.go.kr (J.S.); 24773@seoul.go.kr (S.P.); 6 Korea Armed Forces Nursing Academy, Daejeon 34059, Republic of Korea; ymjungbest@gmail.com; 7 Seoul Infectious Disease Research Center, Seoul Metropolitan Government, Seoul 04524, Republic of Korea

**Keywords:** SARS-CoV-2, COVID-19, homeless, rapid antibody testing, validity

## Abstract

(1) Background: There is a paucity of data regarding the validity of rapid antibody testing for SARS-CoV-2 vaccine response in homeless people worldwide. The objective of this study was to evaluate a rapid SARS-CoV-2 IgM/IgG antibody detection kit as a qualitative screen for vaccination in homeless people. (2) Methods: This study included 430 homeless people and 120 facility workers who had received one of BNT162b2, mRNA-1273, AZD1222/ChAdOx1, or JNJ-78436735/AD26.COV2.5 vaccines. They were tested for IgM/IgG antibodies to the SARS-CoV-2 spike protein with the STANDARD™ Q COVID-19 IgM/IgG Plus Test (QNCOV-02C). ELISA/competitive inhibition ELISA (CI-ELISA) was subsequently run to assess the validity of the serological antibody test. (3) Results: The sensitivity of homeless people was 43.5%. The status of homelessness was related to a lower agreement between serological antibody testing and CI-ELISA (adjusted OR (aOR), 0.35; 95% CI, 0.18–0.70). However, the Heterologous boost vaccine presented higher agreement between serological antibody testing and CI-ELISA (adjusted OR (aOR), 6.50; 95% CI, 3.19–13.27). (4) Conclusions: This study found weak agreement between the rapid IgG results and confirmatory CI-ELISA testing in homeless people. However, it can be used as a screening test for the acceptance of homeless people with heterologous boost vaccination in facilities.

## 1. Introduction

Since it first emerged in Wuhan, China, in December 2019, the coronavirus disease 2019 (COVID-19) epidemic caused by severe acute respiratory syndrome coronavirus 2 (SARS-CoV-2) has progressed rapidly into a pandemic [1]. The coronavirus disease (COVID-19) pandemic is still overwhelming global healthcare systems due to the enormous spread of life-threatening pneumonia that, as of 25 January 2022, has caused 5,832,333 recognized deaths worldwide [2]. The COVID-19 pandemic is ongoing, sustained by the emergence of new variants of concern, such as Omicron (B.1.1.529) and their subvariants. Among those variants, the Omicron variant presents a higher reinfection rate, associated with immune escape [3]. In a previous study, hospitalized patients with respiratory failure due to severe SARS-CoV-2 pneumonia or acute respiratory distress syndrome (ARDS) were found to have a significantly higher morbidity and mortality rate [4]. It is accepted that patients with severe COVID-19 have exhausted antiviral defenses and have an aberrant pulmonary and systemic inflammatory response—also referred to as a “cytokine storm”, the leading cause of organ damage [4,5]. Therefore, evaluating the vaccine’s effectiveness in suppressing the cytokine storm and finding an effective method to measure the antibody is crucial. 

Among other populations, homeless people are at increased risk of SARS-CoV-2 infection [6,7]. Due to the Russian invasion of Ukraine, many Ukrainians are experiencing homelessness. Therefore, they require various types of international help including shelter and livelihood [8]. These situations could result in the increasing risk of transmission of COVID-19 to other countries that are accepting and helping refugees from Ukraine [9]. Therefore, it is essential to understand the serological antibody test for fast, inexpensive, and extensive scale testing and the degree of herd immunity to the COVID-19 vaccination of the homeless [10,11]. However, no articles describing the COVID-19 antibody response to the vaccine among homeless people, nor the validity of the serological antibody test, have been published in any country to date. 

It would be more beneficial to use the rapid antibody test, which is cheaper, less time-consuming, and less invasive, if it has no difference in validity from ELISA antibody measurement after COVID vaccination. In addition, it would be helpful to choose which test to use in group facilities if the difference in validity of different rapid antibody tests according to the type of vaccination and the status of comorbidity in the homeless, one of the most vulnerable populations, can be identified. Therefore, this study aims to evaluate a rapid SARS-CoV-2 IgM/IgG antibody detection kit as a qualitative screen for vaccination in homeless people as a way of suggesting a more affordable and effective screening test for the vulnerable populations. 

## 2. Materials and Methods

### 2.1. Study Design and Subjects

**Figure 1:**
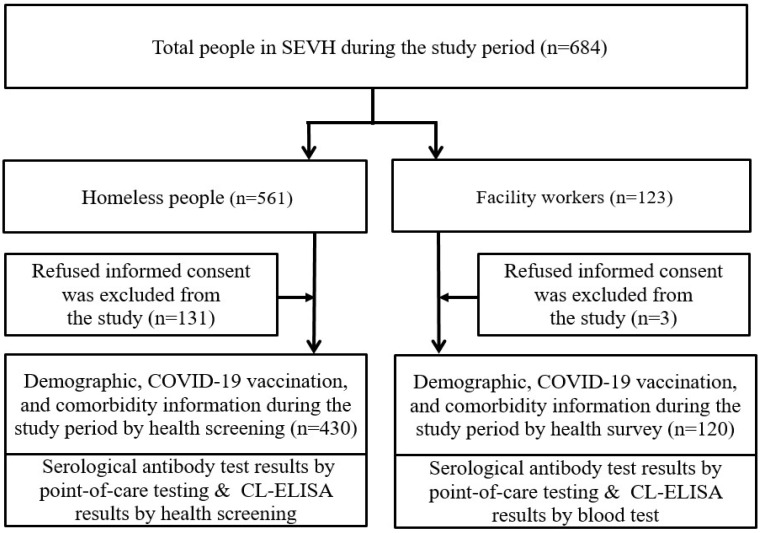
Study population.

The Seoul Eunpyeong Village for Homeless (SEVH) is a Seoul-based non-profit organization that specifically aims to help homeless men by providing shelter, finding their family members, and curing their illness in South Korea. A total of 561 homeless people and 123 staff members (facility workers) in the SEVH were invited to participate in this study from 12 October to 26 November 2021. Any individual who had previously tested positive for SARS-CoV-2 via a reverse transcriptase PCR (RT-PCR, *n* = 0) or not signed informed consent were excluded from the study (*n* = 134). Finally, 550 participants—430 homeless people and 120 workers—were included in this study and all of them were 18 years old or older (Figure 1). 

For those homeless participants, demographic information including sex, age, height, weight, vaccination status and type, and comorbidities, surveyed upon their admission to SEVH, was collected. The researchers visited SEVH during the research period to conduct a serological antibody test as point-of-care testing to determine whether the antibody of the COVID-19 vaccine was present. Moreover, CL-ELISA was performed using the remaining serum after the serological antibody examination. For the participants among the facility workers, the researchers collected data concerning their sex, age, height, weight, vaccination status and type, and comorbidities in a health survey. The serological antibody test and CL-ELISA test were performed for the participating facility workers as well. 

All subjects who participated in the study had received one of four vaccines: BNT162b2, mRNA-1273, AZD1222/ChAdOx1, or JNJ-78436735/AD26.COV2.5. The second dose was taken 50–204 days prior to study enrolment with serological testing, except for the JNJ-78436735/AD26.COV2.5 vaccine, for which the schedule was completed with a single dose. 

### 2.2. COVID-19 Testing 

The nasopharyngeal samples or sputum of the study population were collected in a sterile cup by point-of-care testing. The samples were transferred to Seobuk Hospital in Seoul City, and the institution tested the samples to identify COVID-19 infection cases. For the test, Real-time RT-PCR was performed using an Allplex 2019-nCoV Assay (Seegene, Seoul, Republic of Korea) and a CFX96 Touch Real-Time PCR Detection System (Bio-Rad, Hercules, CA, USA). Those confirm the kinetics of the virus by converting the cycle threshold of Real-time RT-PCR into the number of COVID-19 viral RNA copies. The minimum threshold was set at 2690 copies/mL.

### 2.3. Serological Antibody Testing

The participants’ blood samples were collected from 12 October to 26 November with their informed consent. All samples were tested with the STANDARD™ Q COVID-19 IgM/IgG Plus Test (QNCOV-02C), which obtained authorization for use from the Ministry of Food and Drug Safety on 6 November 2020 (authorization number: 4413). An IgG analysis performed by the manufacturer for 283 samples showed that the rapid test kit had 95.49% sensitivity and 98.67% specificity to anti-spike IgG (Supplement S1). All fingerstick sampling and antibody testing in this study were conducted by trained personnel according to the manufacturer’s instructions. All serum/plasma samples were stored at 4 °C prior to analysis.

**Figure 2:**
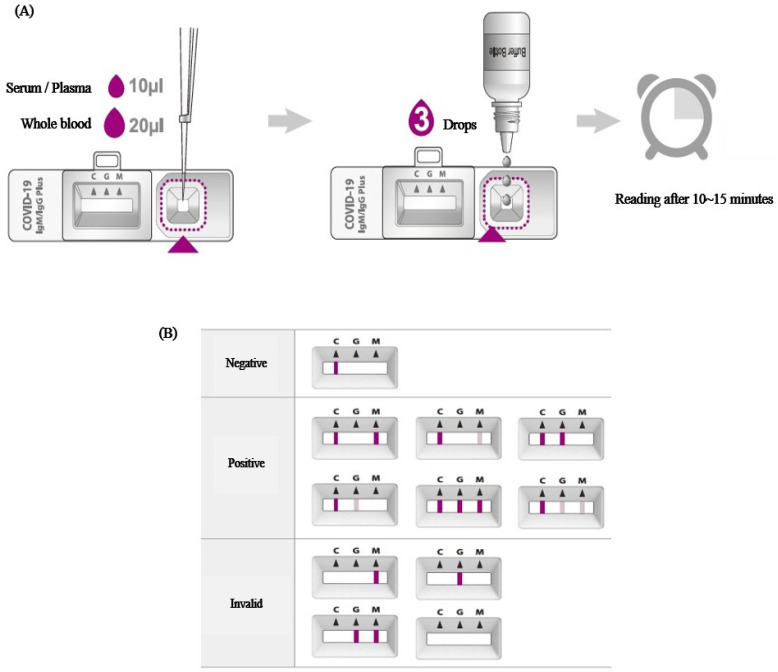
Test procedure (**A**) and results reading guide (**B**) in serologic antibody test.

Rapid antibody test is a test method in which blood samples are dropped into a rapid cassette. In this rapid cassette, (1) the SARS-CoV-2 antigen combined with colloidal gold and (2) Anti-human IgM and IgG, antibodies to IgM and IgG, exist as lines. If IgM or IgG is present in the dropped blood sample, it binds to the anti-IgG/M present in the cassette in the form of invisible lines, and the colloidal gold-covid antigen adheres to the human IgG/IgM in the combined blood, resulting in an observable color change. The anti-IgG/M line will show no change in color without antibodies in the blood sample (Figure 2). 

### 2.4. CL-ELISA

The ADVIA Centaur^®^ SARS-CoV-2 Total (COV2T) assay is for in vitro diagnostic use in the qualitative detection of total antibodies (IgG and IgM), including neutralizing antibodies, to SARS-CoV-2 in human serum and plasma (EDTA and lithium heparin) obtained by venipuncture or capillary puncture using the ADVIA Centaur^®^ XP and ADVIA Centaur^®^ XPT systems. This assay is intended as an aid in identifying patients with an adaptive immune response to SARS-CoV-2. A positive test result may indicate vaccine-derived antibodies to SARS-CoV-2 in vaccinated individuals.

The ADVIA Centaur COV2T assay is a fully automated 1-step antigen sandwich immunoassay using acridinium ester chemiluminescent technology, in which antigens are bridged by antibodies present in the sample. The Solid Phase contains a preformed complex of streptavidin-coated microparticles and biotinylated SARS-CoV-2 recombinant antigens. This reagent is used to capture anti-SARS-CoV-2 antibodies in the sample. The Lite Reagent contains acridinium-ester-labeled SARS-CoV-2 recombinant antigens used to detect anti-SARSCoV-2 antibodies bound to the Solid Phase.

A direct relationship exists between the amount of SARS-CoV-2 antibodies present in the participant sample and the amount of relative light units (RLUs) detected by the system. A result of reactive or nonreactive is determined according to the Index Value established with the calibrators. The system reports ADVIA Centaur COV2T assay results in Index Values or U/mL and as nonreactive or reactive: (1) Nonreactive: <1.00 Index (U/mL); these samples are considered negative for SARS-CoV-2 antibodies. (2) Reactive: ≥1.00 Index (U/mL); these samples are considered positive for SARS-CoV-2 antibodies. Results of this assay should always be interpreted in conjunction with the participant’s medical history, clinical presentation, and other findings.

The ADVIA Centaur COV2T assay standardization is traceable to an internal standard based on agreement with known positive and negative SARS-CoV-2 samples. The internal standardization supports reporting of results in Index Values or U/mL. The analytical sensitivity at the cut-off values for the ADVIA Centaur COV2T assay was determined on the ADVIA Centaur XP system using the World Health Organization (WHO) 1st International Standard for anti-SARS-CoV-2 immunoglobulin (human) NIBSC code: 20/136. The concentration of the reference standard that corresponds to the cut-off value of 1.00 Index (U/mL) for the ADVIA Centaur COV2T assay is 6.57 BAU/mL (Supplement S2).

### 2.5. Statistical Analysis

Statistical analysis was performed using SPSS Software for Windows, version 12.0 (SPSS Inc., Chicago, IL, USA). To validate the serological antibody testing, we examined its sensitivity, specificity, positive predictability, and accuracy in the data. All reported *p*-values were two-sided and considered statistically significant as *p*-value < 0.05. We established a logistic regression model to verify the testing agreement between rapid serological antibody testing and the chemiluminescent immunoassay. To evaluate the adequacy of each model, we used a Hosmer–Lemeshow test in which we considered the goodness-of-fit of each model if the *p*-value was >0.05. Nagelkerke’s R^2^ was used to define the power of explanation of the logistic regression model. Odds ratios were calculated using 95% confidence intervals (CIs).

## 3. Results

### 3.1. Classification of COVID-19 Vaccines in Study Population

The differences in boosting vaccination between the samples of homeless people and facility workers were as follows. The homeless sample had a higher boost vaccination rate than the other: 419 out of 430 homeless people (97.4%); 110 out of 120 facility workers (91.7%). There was also a difference in the ratio of the type of boost vaccine and homologous vaccination. AZD1222/ChAdOx1 was the most frequently used vaccine for the initial and the boost vaccinations in both groups in homologous vaccination. However, the inoculation rate was different: 86% (370 out of 430) for the homeless group and 46.7% (56 out of 120) for the other group. On the other hand, AZD1222/ChAdOx1 for the first round and BNT162b2 for the second round was the most common combination in the heterologous vaccination. The vaccination rate in the homeless sample was 10.7% (46 persons), lower than the 29.2% (35 persons) in the facility worker group (Table 1).

### 3.2. Validity of Serological Rapid Testing in Study Population

The baseline characteristics of the study participants are presented in Table 2. Of the total 550 participants, 273 (49.6%) tested positive for IgG to the CL-ELISA on the serological antibody test and one participant (0.18%) who tested IgG-negative via the serological antibody test was positive on anti-spike IgG CI-ELISA testing. No participant was found to be IgM-positive via the serological antibody test. The sensitivity of homeless people (43.5%) was lower than that of facility workers (74.2%). The most sensitive indicator for detection of the serological antibody test was heterologous boost vaccination of COVID-19 with 88% sensitivity. On the other hand, a Charlson comorbidity index (CCI) score ≥3 was the least sensitive indicator (29.2%). Especially, the sensitivities of liver cirrhosis and kidney were lower than 20% (Table 3, Supplement S3). The specificity of most indicators was over 80%, except for the CCI score with 50% (Table 2, Supplement S3). 

### 3.3. Comparisons of Validity of Serological Antibody Testing between Homeless People and Facility Workers

The sensitivity of serological antibody testing in homeless people was lower than in facility workers by vaccination status and comorbidity: homologous vaccination (38.0% vs. 67.9%), heterologous vaccination (87.2% vs. 88.9%), non-comorbidity (57.3% vs. 77.6%), and comorbidity (40.1% vs. 68.2%). The specificity ranged from 85.7% to 100% in homeless people (Table 4, Supplement S3).

### 3.4. Agreement Level for Validity between Serological Antibody Testing and CL-ELISA

The agreement level and adjusted odds ratios (aOR) for validity between serological antibody testing and CL-ELISA are summarized in Table 4. Heterologous booster vaccination was associated with a high agreement level of serological rapid testing for total participants (aOR, 6.50; 95% CI, 3.19~13.27) and homeless people (aOR, 8.86; 95% CI, 3.58~21.92); homeless people were associated with a low agreement factor of serological antibody testing (aOR, 0.35; 95% CI, 0.18~0.70) (Table 5).

## 4. Discussion

In this study, we evaluated the validity of a serological antibody test to screen for a vaccine-mediated SARS-CoV-2 antibody response in homeless people. The boost vaccination rate of the homeless people, who belonged to SEVH, was 97.4%, higher than 91.7% of the facility workers. Despite the higher rate of overall vaccination, the homeless sample presented a lower rate in heterologous vaccination—known as having a stronger immunity enhancement—than the facility worker sample. On top of that, the higher comorbidity rate and age would have reduced the effectiveness of the vaccines. It was found that the serological antibody test results were correlated weakly with confirmatory CI-ELISA testing in the homeless. The positive IgG serology in the homeless and the facility workers—43.5% and 74.2%, respectively—in this study were different from previous studies investigating the immunogenicity of BNT162b2 and mRNA-1273 involved in this study as well. Heyming et al. demonstrated that 98.4% of pediatric healthcare workers (*n* = 125) were IgG-positive and 0.8% of them were IgM-positive on rapid antibody testing [11]. These differences may have been caused by the fact that Heyming et al. recruited healthy people aged 18 or over who were providing medical care to COVID-19 patients. Another difference was that the participants had ideal conditions for such a study as they received two doses of either BNT162b2 or mRNA-1273 vaccine and the second dose was taken 17~36 days before the study enrolment. However, in a real-world sample, such as homeless people who are vulnerable to infectious disease with lower immunity level and with mixed intervals between the first and second doses ranging from one month to six months, the accuracy of the serological antibody test could be lower, as shown in this study. 

On the other hand, Horie et al. [10] reported that among 173 workers assisting the homeless during the COVID-19 pandemic, 26% tested positive in the serological antibody test; further, two (1.6%) out of one hundred and seventy-three homeless people tested IgM-positive. In another study by Ralli et al. [12], one (0.6%) person tested IgG-positive. Compared with the result of this study, however, such results in previous studies may be considered underestimated. 

Nevertheless, it is important to identify the influencing factors of the validity of serological antibody testing. This study found that antibody positivity rate was different depending on the status of homelessness, sex, age, BMI, heterologous vaccination, and comorbidity. Among these factors, as the comorbidity score increased, the sensitivity decreased. In this study, the sensitivity of the rapid antibody test results after COVID-19 vaccination in diabetic patients was 34.3%. Based on the results of previous studies, which founded that the risk of ARDS increased in COVID-19 patients with comorbidities including diabetes, it can be presumed that the vaccine’s immunizing effect may be reduced and the risk of cytokine storm may be increased [2,4]. Especially, for participants with liver cirrhosis or kidney diseases such as chronic renal failure, the sensitivity decreased to 20% or lower. A previous study reported poor antibody responses after SARS-CoV-2 vaccination in 61% of liver transplant recipients and 24% of those with chronic liver diseases [13]. Based on this evidence, a possible explanation is that vaccination could have lowered the immunity level in participants with comorbidities and, thus, their test results were false-negative in this study. 

Heterologous boost vaccination and homeless status were associated with agreement between serological antibody tests and CL-ELISA after adjusted logistic regression analysis because heterologous boost vaccination reported greater reactogenicity following prime vaccination, comparable to homologous boosting [14,15,16,17,18].

Although this study is the first to assess validity of serological antibody testing in homeless people, this study has two limitations. First, it is a cross-sectional design study. Therefore, it is impossible to examine the changes in antibody positivity rate by time flow. Second, as the homeless participants were all males, this study is not able to identify the validity of serological antibody testing in the female homeless population. 

## 5. Conclusions

This study found weak agreement between the rapid IgG results and confirmatory CI-ELISA testing in homeless people. In addition, there was a difference in concordance rates depending on homelessness and heterologous boost vaccination of COVID-19. Nevertheless, a rapid antibody test can be chosen as a quick screening test for the homeless to verify their level of immunity and to determine whether to accommodate the homeless people with heterologous boost vaccination in facilities. To overcome the limitations of this study, we suggest additional studies with, for example, longitudinal designs and/or female homeless samples.

## Figures and Tables

**Table 1:** Classification of COVID-19 vaccines in study population.

	N (%)		Homeless People	Facility Worker	*p*-Value **
Boost vaccine *	No		11 (2.6)	10 (8.3)	0.007
	Yes		419 (97.4)	110 (91.7)	
	First vaccination	Second vaccination	Homeless people	Facility worker	
	JNJ-78436735/AD26.COV2.5		5 (1.2)	7 (5.8)	<0.001
	AZD1222/ChAdOx1		6 (1.4)	3 (2.5)	
	AZD1222/ChAdOx1	AZD1222/ChAdOx1	370 (86)	56 (46.7)	
	AZD1222/ChAdOx1	BNT162b2	46 (10.7)	35 (29.2)	
	mRNA-1273	BNT162b2	1 (0.2)	0 (0)	
	mRNA-1273	mRNA-1273	1 (0.8)	1 (0.2)	
	BNT162b2	AZD1222/ChAdOx1	0 (0)	1 (0.8)	
	BNT162b2	BNT162b2	1 (0.2)	17 (14.2)	

*: mRNA vaccine: mRNA-1273 and BNT162b2. Vector vaccine: AZD1222/ChAdOx1 and JNJ-78436735/AD26.COV2.5. **: χ^2^ test and Fisher’s exact test (*p*-value < 0.05).

**Table 2:** Comparisons for validity of serological antibody test in study population *.

	Indicator	N (%)	Sensitivity	Specificity	Positive Predictability	Accuracy
	Total people	550 (100)	50.3	88.9	99.6	50.9
	Homeless people	430 (78.2)	43.5	88.9	99.5	44.4
	Facility worker	120 (21.8)	74.2		100	
Sex	Female	49 (8.9)	65.3		100	
	Male **	501 (91.1)	48.8	88.9	99.6	49.5
Age ***	<65 yrs	369 (67.1)	57.7	100	100	58.0
	≥65 yrs	180 (32.7)	35.1	83.3	98.4	36.7
BMI ***	18.5~29.99	477 (86.7)	50.9	88.9	99.6	51.6
	<18.5	39 (7.1)	38.5		100	
	≥30	32 (5.8)	53.1		100	
Heterologous vaccination	No	467 (84.9)	43.3	88.9	99.5	44.3
	Yes	83 (15.1)	88.0		100	
Comorbidity	No	160 (29.1)	50.3	88.9	99.6	51.6
	Yes	390 (70.9)	38.5		100	
CCI ***	0	294 (53.5)	60.5	100	100	60.9
	1	133 (24.2)	42.7	50	98.2	42.9
	2	74 (13.5)	36.3	100	100	39.2
	≥3	49 (8.9)	29.2		100	

* Gold standard test was chemiluminescent immunoassay, CLIA. ** Homeless people were all male. *** Homeless people have a missing value of age (*n* = 1). Facility workers have a missing value of BMI (*n* = 2) and those aged 65 or older were only homeless people.

**Table 3:** Comparisons for validity of serological antibody test in study population by comorbidity.

	Indicator	N (%)	Sensitivity	Specificity	Positive Predictability	Accuracy
Hypertension	No	360 (65.5)	55.2	85.7	99.5	55.8
	Yes	190 (34.5)	41.0	100	100	53.2
Diabetes mellitus	No	477 (86.7)	52.7	100	100	53.2
	Yes	73 (13.3)	34.3	66.7	96.0	35.6
Diabetes mellitus and End-organ disease	No	547 (99.5)	50.4	88.9	99.6	51.0
	Yes	3 (0.5)	33.3		100	
Lipidemia	No	478 (86.9)	50.7	88.9	99.6	51.5
	Yes	82 (13.1)	47.2		100	
Cancer	No	527 (95.8)	51.2	88.9	99.6	51.8
	Yes	23 (4.2)	30.4		100	
Mild liver disease	No	512 (93.1)	50.7	88.9	99.6	51.4
	Yes	38 (6.9)	44.7		100	
Moderate or severe liver disease	No	545 (99.1)	50.6	88.9	99.6	51.2
	Yes	5 (0.9)	20		100	
Connective tissue disease	No	495 (90)	51.5	87.5	99.6	52.1
	Yes	55 (10)	38.9	100	100	40.0
Cerebrovascular accident	No	525 (95.5)	50.9	87.5	99.6	51.4
	Yes	25 (4.5)	37.5	100	100	40.0
Hemiplegia	No	528 (96)	51.1	88.9	99.6	51.7
	Yes	22 (4)	31.8		100	
Gastrointestinal ulcer disease	No	484 (88)	52.1	87.5	99.6	52.7
	Yes	66 (12)	36.9	100	100	37.9
Myocardial infarction	No	540 (98.2)	50.4	87.5	99.6	50.9
	Yes	10 (1.8)	44.4	100	100	50.0
Moderate to severe renal disease	No	542 (98.5)	50.7	85.7	99.6	51.1
	Yes	8 (1.5)	16.7	100	100	37.5
Chronic pulmonary disease	No	520 (94.5)	50.6	87.5	99.6	51.2
	Yes	30 (5.5)	44.8	100	100	46.7
Psychiatric disease	No	517 (94)	51.1	87.5	99.6	51.6
	Yes	33 (6)	37.5	100	100	39.4
Urologic disease	No	456 (82.9)	53.6	83.3	99.6	53.9
	Yes	94 (17.1)	34.1	100	100	36.2
Ophthalmological disease	No	525 (95.5)	50.2	88.9	99.6	50.9
	Yes	25 (4.5)	52.0		100	
Endocrine disease (except for diabetes mellitus)	No	541 (98.4)	50.5	87.5	99.6	51
	Yes	9 (1.6)	37.5	100	100	44.4
Dermatological disease	No	545 (99.1)	50.2	88.9	99.6	50.8
	Yes	5 (0.9)	60		100	
Miscellaneous disease	No	494 (89.8)	52.7	87.5	99.6	53.2
	Yes	56 (10.2)	29.1	100	100	30.4

**Table 4:** Comparisons for validity of serological antibody test between homeless people and facility worker by cross vaccine and comorbidity.

	**Indicator**	**N (%)**	**Sensitivity**	**Specificity**	**Positive** **Predictability**	**Accuracy**
	Total	550 (100)	50.3	88.9	99.6	50.9
	Homeless people	430 (78.2)	43.5	88.9	99.5	44.4
	Facility workers	120 (21.8)	74.2		100	
Homologous vaccination	Total	467 (84.9)	43.3	88.9	99.5	44.3
	Homeless people	383 (69.6)	38.0	88.9	99.3	39.2
	Facility workers	84 (15.3)	67.9		100	
Heterologous vaccination	Total	83 (15.1)	88.0		100	
	Homeless people	47 (8.5)	87.2		100	
	Facility workers	36 (6.5)	88.9		100	
Non-comorbidity	Total	160 (29.1)	67.1	100	100	67.5
	Homeless people	84 (15.3)	57.3	100	100	58.3
	Facility workers	76(13.8)	77.6		100	
Comorbidity	Total	390 (70.9)	43.3	85.7	99.4	44.1
	Homeless people	346 (62.9)	40.1	85.7	99.3	41.0
	Facility workers	44 (8)	68.2		100	

**Table 5:** Adjusted odds ratios of agreement between serological antibody test and chemiluminescent immunoassay *.

	Indicator	Total	Facility Workers	Homeless People
Sex	Female	1	1	
	Male	1.81 (0.76–4.31)	1.67 (0.68–4.09)	
Age	<65 yrs	1		1
	≥65 yrs	0.85 (0.56–1.29)		0.87 (0.57–1.32)
BMI	18.5~29.99	1	1	1
	<18.5	0.66 (0.32–1.36)	0.92 (0.07–12.37)	0.62 (0.29–1.34)
	≥30	1.03 (0.47–2.26)		0.80 (0.33–1.92)
Heterologous vaccination	No	1	1	1
	Yes	6.50 (3.19–13.27)	3.17 (0.98–10.23)	8.86 (3.58–21.92)
Homeless	No	1		
	Yes	0.35 (0.18–0.70)		
CCI	0	1	1	1
	1	0.64 (0.40–1.01)	1.05 (0.25–4.48)	0.62 (0.38–1.01)
	2	0.68 (0.38–1.19)	0.51 (0.03–8.68)	0.69 (0.39–1.24)
	≥3	0.53 (0.27–1.05)		0.53 (0.26–1.06)
Hosmer–Lemeshow test, χ^2^ (*p*-value)		5.06 (0.69)	1.58 (0.90)	5 (0.54)
Nagelkerke’s R^2^		0.20	0.14	0.15

* Odds ratios (95% CI) estimated by logistic regression controlling for sex, age, BMI, cross vaccine, homelessness, and CCI with statistical significance are presented in boldface.

## Data Availability

The data presented in this study are available in Supplement S3.

## Supplementary Materials

The following supporting information can be downloaded at: https://www.mdpi.com/article/10.3390/v15061400/s1, Supplement S1: Information of the STANDARD™ Q COVID-19 IgM/IgG Plus Test (QNCOV-02C); Supplement S2: Information on the ADVIA Centaur XP and ADVIA Centaur XPT Systems; Supplement S3: Data of validity.
