# Supplementary material for: Validity of Rapid Antibody Testing for COVID-19 Vaccine in Homeless People
Source: Viruses. 2023 Jun 20;15(6):1400. doi: 10.3390/v15061400 (PMC10305635; doi:10.3390/v15061400)
Supplement: Supplementary file 1 [file viruses-15-01400-s001.zip › Supplement S1.pdf]

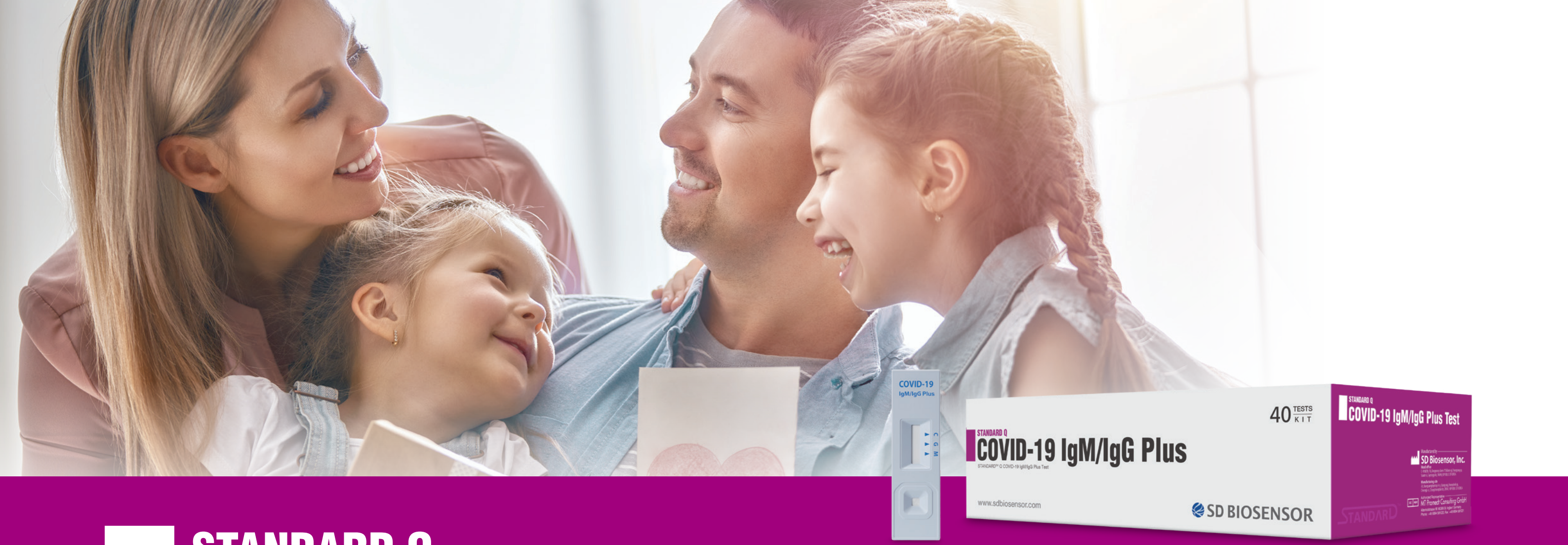

STANDARD Q
COVID-19 IgM/IgG Plus Test

- 국내 최초 KFDA 내수용 허가 승인
- 10-15분 이내 빠른 검사 결과 확인
- SARS-CoV-2 항체 신속 진단
- 집단 수준에서의 혈청학적 검사에 사용될 수 있음
- anti-NP, anti-SP 항체 진단가능
- RT-PCR의 검출 한계 이하의 SARS-CoV-2 에 감염된 이력이 있는 환자를 진단하는데 유용

- 쉽고 간편한 사용
- 97.74%의 민감도
- 98.67%의 특이도

CE IVD
KOREA MFDS Approved

[감염 경과에 따른 사용 적합 시기]

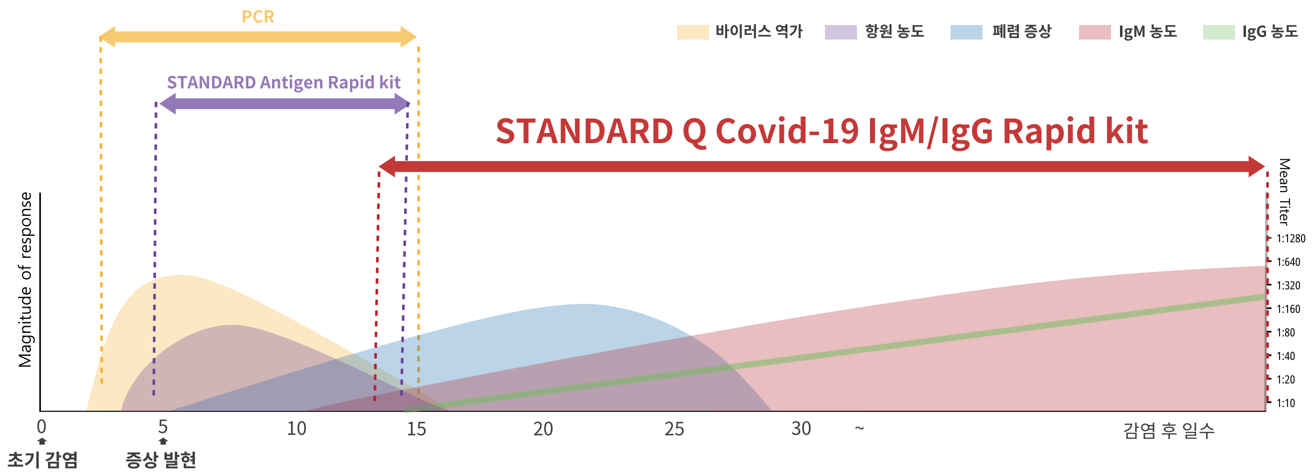

	항체 검사	항원 검사	분자진단 검사
검사 목적	COVID-19 바이러스에 대한 **항체 생성여부 확인**	COVID-19 바이러스 **특정 단백질 유무 확인**	COVID-19 바이러스 **유전자 유무 확인**
사용 검체	혈액	코 또는 목의 점액	코 또는 목의 점액, 가래 (객담)
검사 시간	약 15분	약 30분	약 3-6시간
장점	**과거 감염이력 확인 가능 검사시간 짧고 비용 낮음**	**유전자 검사 대비 검사 시간 짧고 비용 낮음**	**정확도가 높아 확진용으로 사용**
자사제품	STANDARD Q COVID-19 항체 검사	STANDARD Q COVID-19 항원 검사	STANDARD M nCoV 분자진단검사

표 내용 출처 : 식품안전의약처

[그래프 내용 출처]

1. Cellular immune responses to severe acute respiratory syndrome coronavirus infection in senescent BALB/c Mice:CD4+ T cells are important in control of SARD-CoV infection.
2. Chronological evolution of IgM, IgA, IgG and neutralization antibodies after infection with SARD-Associated coronavirus.
3. Mechanisms of Host defense following severe acute respiratory syndrome-coronavirus pulmonary infection of mice.
4. SARS immunity and vaccination.

SD BIOSENSOR

검사 절차

1 검체 (혈청/혈장/전혈) 채취 후 검체 점적부위에 점적

2 버퍼 3 방울 점적

3 10-15분 후 결과 확인

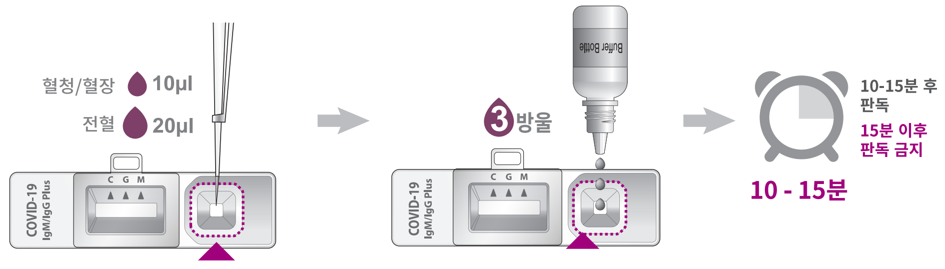

결과 해석

음성	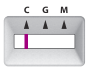
양성	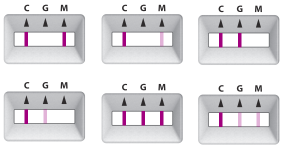
재시험	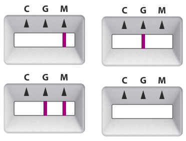

임상 결과

임상적 민감도 및 임상적 특이도

본 임상적 성능시험과 관계없이 코로나19가 의심되어 임상 평가 기관에 내원하고 긴급사용승인된 RT-PCR로 양성으로 확인된 환자에서 채취한 잔여 혈청 검체 133개와 음성으로 확인된 잔여검체 150개를 대상으로 신청제품의 임상적 민감도 및 임상적 특이도를 확인한 결과는 다음과 같습니다.

1) IgM+IgG 결과 (*IgM과 IgG 중 하나라도 양성일 경우 양성으로 판정)

		긴급사용승인된 RT-PCR 시험결과		
		Positive	Negative	Total
STANDARD Q COVID-19 IgM/IgG Plus	Positive	130	2	132
	Negative	3	148	151
	Total	133	150	283
임상적 민감도 (Sensitivity)		97.74% (130/133) [95% 신뢰구간: 93.55% - 99.53%]		
임상적 특이도 (Specificity)		98.67% (148/150) [95% 신뢰구간: 95.27% - 99.84%]		

2) IgM 결과

		긴급사용승인된 RT-PCR 시험결과		
		IgM Positive	IgM Negative	Total
STANDARD Q COVID-19 IgM/IgG Plus	Positive	125	0	125
	Negative	8	150	158
	Total	133	150	283
임상적 민감도 (Sensitivity)		93.98% (125/133) [95% 신뢰구간: 88.49% - 97.37%]		
임상적 특이도 (Specificity)		100.00% (150/150) [95% 신뢰구간: 97.57% - 100.00%]		

3) IgG 결과

		긴급사용승인된 RT-PCR 시험결과		
		IgG Positive	IgG Negative	Total
STANDARD Q COVID-19 IgM/IgG Plus	Positive	127	2	129
	Negative	6	148	154
	Total	133	150	283
임상적 민감도 (Sensitivity)		95.49% (127/133) [95% 신뢰구간: 90.44% - 98.33%]		
임상적 특이도 (Specificity)		98.67% (148/150) [95% 신뢰구간: 95.27% - 99.84%]		

검체 채취기간별 임상적 민감도

본 임상적 성능시험과 관계없이 코로나19가 의심되어 임상 평가 기관에 내원하고 긴급사용승인된 RT-PCR로 양성으로 확인된 환자에서 채취한 잔여 혈청 검체 133개를 대상으로 검체 채취 기간별 임상적 민감도를 분석한 결과는 다음과 같습니다.

1) IgM+IgG 결과

증상발현 후 검체 채취 시점까지 기간	민감도
< 7일	78.6% (11/14)
7 ~ 14일	100.0%(30/30)
> 14일	100%(89/89)

2) IgM 결과

증상발현 후 검체 채취 시점까지 기간	민감도
< 7일	78.6% (11/14)
7 ~ 14일	100%(30/30)
> 14일	94.4%(84/89)

3) IgG 결과

증상발현 후 검체 채취 시점까지 기간	민감도
< 7일	78.6% (11/14)
7 ~ 14일	96.7%(29/30)
> 14일	97.8%(87/89)

*IgM과 IgG 중 하나라도 양성일 경우 양성으로 판정

주문 정보

Cat No.	제품	Test/ Kit	Kit/ Carton
09COV70G	STANDARD Q COVID-19 IgM/IgG Plus Test	40	40

STANDARD™ www.sdbiosensor.com

LEF-Q-NCOV-02C -KR_Rev.00 / 202011

Head Office C-4&5Floor, 16, Deogyeong-daero 1556beon-gil, Yeongtong-gu, Suwon-si, Gyeonggi-do, 16690, REPUBLIC OF KOREA
Tel +82-31-300-0400 | Fax +82-31-300-0499 | E-mail sales@sdbiosensor.com

© 2020 SD BIOSENSOR. All rights reserved.

SD BIOSENSOR
