# Supplementary material for: Validity of Rapid Antibody Testing for COVID-19 Vaccine in Homeless People
Source: Viruses. 2023 Jun 20;15(6):1400. doi: 10.3390/v15061400 (PMC10305635; doi:10.3390/v15061400)
Supplement: Supplementary file 1 [file viruses-15-01400-s001.zip › Supplement S2.pdf]

SIEMENS

ADVIA Centaur® XP
ADVIA Centaur® XPT
Immunoassay Systems

SARS-CoV-2 Total (COV2T)

Assay for the Detection of Total Antibodies to SARS-CoV-2

**Current Revision and Date**[a]	Rev. 03, 2021-05	
**Product Name**	ADVIA Centaur SARS-CoV-2 Total (COV2T)	REF 11206710 (100 tests)<br>REF 11206922 (500 tests)
**Abbreviated Product Name**	ADVIA Centaur COV2T	
**Test Name/ID**	COV2T	
**Systems**	ADVIA Centaur XP system<br>ADVIA Centaur XPT system	
**Materials Required but Not Provided**	ADVIA Centaur Probe Wash 3	REF 03333963
	ADVIA Centaur Wash 1 (2 x 1500 mL)	REF 01137199 (112351)
	ADVIA Centaur Wash 1 (2 x 2500 mL)	REF 03773025
**Optional Materials**	ADVIA Centaur COV2T QC	REF 11206713
	ADVIA Centaur Multi-Diluent 2	REF 07948423 (110314)
	ADVIA Centaur COV2T MCM	REF 11207583
**Specimen Types**	Serum, EDTA plasma, lithium heparin plasma (venipuncture or capillary puncture)	
**Sample Volume**	50 µL	
**Measuring Interval**	0.60–45.00 Index (U/mL)	

[a] A vertical bar in the page margin indicates technical content that differs from the previous version.

CE

Intended Use

The ADVIA Centaur® SARS-CoV-2 Total (COV2T) assay is for *in vitro* diagnostic use in the qualitative and quantitative detection of total antibodies (IgG and IgM), including neutralizing antibodies, to SARS-CoV-2 in human serum and plasma (EDTA and lithium heparin) obtained by venipuncture or capillary puncture using the ADVIA Centaur® XP and ADVIA Centaur® XPT systems.

This assay is intended as an aid in the diagnosis of patients with suspected SARS-CoV-2 infection and as an aid in identifying patients with an adaptive immune response to SARS-CoV-2, indicating recent or prior infection. A positive test result may indicate vaccine-derived antibodies to SARS-CoV-2 in vaccinated individuals.

Summary and Explanation

COVID-19 (coronavirus disease 2019) is the illness resulting from infection with SARS-CoV-2 (severe acute respiratory syndrome coronavirus 2) virus.[1-5] The virus spreads readily from person to person or possibly from environmental exposure.[6] Evidence supports spread by both asymptomatic and symptomatic individuals.[7] Some studies reported that about 20% of infections produce severe disease, principally Acute Respiratory Distress Syndrome (ARDS), requiring intensive care unit treatment.[4,8,9] Differentiating COVID-19 from other respiratory pathogens is essential for implementing infection control measures, such as isolation and contact tracing, as well as clinical monitoring and support.

Diagnosis of current infection with SARS-CoV-2 relies primarily on molecular testing for the viral RNA using a swab collection for sputum or throat/nasal secretions.[10,11] SARS-CoV-2 RNA testing is recommended as the most sensitive diagnostic test for early infection, as viral RNA can be detected prior to antibody seroconversion.[12,13] However, molecular testing for viral RNA can miss infections due to factors such as insufficient viral load, compromised sample collecting/handling or RNA extraction, and limitations on test sensitivity. Production of antibodies to the virus (such as IgM and IgG) occurs within 15 days in most patients, and seroconversion can be coincident with the continued detection of viral RNA.[13-16] Utilizing a combined approach of both serology (serum or plasma) and RNA testing offers the highest diagnostic sensitivity.[13] Additional data show that use of a total antibody serology test (combined IgM and IgG detection) may provide earlier detection than either IgM or IgG alone.[13,14]

Along with supporting a diagnosis of COVID-19, serology testing is essential for disease surveillance. This is particularly true for understanding viral prevalence, as most infections cause mild or no symptoms. Assessment of antibodies to SARS-CoV-2 virus in the population aids in the understanding of disease prevalence and can support assessment of immunity once antibody-mediated protection is better defined.[17]

Principles of the Procedure

The ADVIA Centaur COV2T assay is a fully automated 1-step antigen sandwich immunoassay using acridinium ester chemiluminescent technology, in which antigens are bridged by antibodies present in the patient sample. The Solid Phase contains a preformed complex of streptavidin-coated microparticles and biotinylated SARS-CoV-2 recombinant antigens. This reagent is used to capture anti-SARS-CoV-2 antibodies in the patient sample. The Lite Reagent contains acridinium-ester-labeled SARS-CoV-2 recombinant antigens used to detect anti-SARS-CoV-2 antibodies bound to the Solid Phase.

A direct relationship exists between the amount of SARS-CoV-2 antibodies present in the patient sample and the amount of relative light units (RLUs) detected by the system.

A result of reactive or nonreactive is determined according to the Index Value established with the calibrators. Refer to *Interpretation of Results*.

Reagents

Material Description	Storage	Stability
**ADVIA Centaur COV2T ReadyPack® primary reagent pack**[a, b]<br>**Lite Reagent**<br>10.0 mL/reagent pack<br>Recombinant SARS-CoV-2 S1 RBD antigen (~0.3 µg/mL) labeled with acridinium ester in buffer; bovine serum albumin; goat serum; surfactant; sodium azide (< 0.1%)<br>**Solid Phase**<br>10.0 mL/reagent pack<br>Streptavidin-coated paramagnetic microparticles preformed with biotinylated SARS-CoV-2 S1 RBD antigen (~1.0 µg/mL) in buffer; bovine serum albumin; goat serum; surfactant; sodium azide (< 0.1%)	Unopened at 2–8°C<br><br>Onboard	Until expiration date on product<br><br>28 days
**ADVIA Centaur COV2T CAL**[a, b]<br>1.0 mL/vial<br>Processed* human plasma nonreactive for antibodies to SARS-CoV-2 and processed* human plasma spiked with antibodies to SARS-CoV-2; sodium azide (< 0.1%)<br>**Processed plasma is defibrinated and filtered plasma.*	Unopened at 2–8°C<br><br>Opened at 2–8°C<br><br>At room temperature	Until expiration date on product<br><br>60 days<br><br>8 hours
**ADVIA Centaur Multi-Diluent 2**[a, b, c]<br>10.0 mL/reagent pack<br>Goat serum; sodium azide (< 0.1%); preservatives	At 2–8°C<br><br>Onboard	Until expiration date on product<br><br>28 days
**ADVIA Centaur Probe Wash 3**[a, d]<br>50.0 mL/pack<br>Sodium hypochlorite (0.5%); sodium hydroxide (< 0.5%); pH 11.0	Unopened at 2–8°C<br><br>Onboard	Until expiration date on product<br><br>100 days
**ADVIA Centaur Wash 1**[a, d]<br>1500 mL/pack<br>Phosphate-buffered saline; sodium azide (< 0.1%); surfactant	Unopened at 2–25°C<br><br>Onboard	Until expiration date on product<br><br>1 month
**ADVIA Centaur Wash 1**[a, d]<br>2500 mL/pack<br>Phosphate-buffered saline; sodium azide (< 0.1%); surfactant	Unopened at 2–25°C<br><br>Onboard	Until expiration date on product<br><br>1 month

[a] Store in an upright position.
[b] Prevent exposure to sunlight and heat.
[c] Refer to *Optional Materials*.
[d] Refer to *Materials Required but Not Provided*.

Warnings and Precautions

For *in vitro* diagnostic use.

For Professional Use.

Safety data sheets (SDS) available on siemens-healthineers.com.

CAUTION POTENTIAL BIOHAZARD

Contains human source material. Each donation of human blood or blood component was tested by FDA-approved methods for the presence of antibodies to human immunodeficiency virus type 1 (HIV-1) and type 2 (HIV-2), as well as for hepatitis B surface antigen (HBsAg) and antibody to hepatitis C virus (HCV). The test results were negative (not repeatedly reactive). No test offers complete assurance that these or other infectious agents are absent; this material should be handled using good laboratory practices and universal precautions.[18-20]

CAUTION

This device contains material of animal origin and should be handled as a potential carrier and transmitter of disease.

Contains sodium azide as a preservative. Sodium azide can react with copper or lead plumbing to form explosive metal azides. On disposal, flush reagents with a large volume of water to prevent buildup of azides. Disposal into drain systems must be in compliance with prevailing regulatory requirements.

Dispose of hazardous or biologically contaminated materials according to the practices of your institution. Discard all materials in a safe and acceptable manner and in compliance with prevailing regulatory requirements.

Storage and Stability

Store all reagents in an upright position, away from light and heat. Do not use products beyond the expiration date printed on the product labeling.

For information about product storage and stability, refer to *Reagents*.

Onboard Stability

Discard products at the end of the onboard stability interval. Do not use products beyond the expiration date printed on the product labeling.

For information about product onboard stability, refer to *Reagents*.

Specimen Collection and Handling

Serum and plasma (EDTA and lithium heparin) are the recommended sample types for this assay. Do not use heat-inactivated specimens.

Collecting the Specimen

- Observe universal precautions when collecting specimens. Handle all specimens as if they are capable of transmitting disease.[20]
- Follow recommended procedures for collection of diagnostic blood specimens by venipuncture or capillary puncture.[21,22]
- Follow the instructions provided with your specimen collection device for use and processing.[23]
- Allow blood specimens to clot completely before centrifugation.[19]
- Keep tubes capped at all times.[19]

Storing the Specimen

- Samples are stable for up to 24 hours onboard the system.
- Separated samples are stable for up to 3 days at room temperature, and for up to 5 days at 2–8°C.

- Thawed frozen specimens must be clarified by centrifugation prior to testing. Do not store in a frost-free freezer. Avoid more than 3 freeze-thaw cycles.
- Freeze samples, devoid of red blood cells, at ≤ -20°C for longer storage.

The handling and storage information provided here is based on data or references maintained by the manufacturer. It is the responsibility of the individual laboratory to use all available references and/or its own studies when establishing alternate stability criteria to meet specific needs.

Transporting the Specimen

Package and label specimens for shipment in compliance with applicable federal and international regulations covering the transport of clinical specimens and etiological agents.

If shipment is expected to exceed 5 days, ship specimens frozen. Store samples capped and upright at 2–8°C upon arrival.

Preparing the Samples

This assay requires 50 µL of sample for a single determination. This volume does not include the unusable volume in the sample container or the additional volume required when performing duplicates or other tests on the same sample. For a complete list of appropriate sample containers and information about determining the minimum required volume, refer to the system online help.

The sample volume required to perform onboard dilution differs from the sample volume required to perform a single determination on an undiluted sample. Refer to *Dilutions*.

Do not use samples with apparent contamination.

Before placing samples on the system, ensure that samples are free of:

- Bubbles or foam.
- Fibrin or other particulate matter.

Remove particulates by centrifugation according to CLSI guidance and the collection device manufacturer's recommendations.[19]

Procedure

Materials Provided

The following materials are provided:

REF	Contents	Number of Tests
11206710	1 ReadyPack primary reagent pack containing ADVIA Centaur COV2T Lite Reagent and Solid Phase<br>ADVIA Centaur COV2T master curve card<br>1 vial ADVIA Centaur COV2T CAL low calibrator CAL L<br>1 vial ADVIA Centaur COV2T CAL high calibrator CAL H<br>ADVIA Centaur COV2T CAL calibrator assigned value sheets and barcode labels	100
11206922	5 ReadyPack primary reagent packs containing ADVIA Centaur COV2T Lite Reagent and Solid Phase<br>ADVIA Centaur COV2T master curve card<br>2 vials ADVIA Centaur COV2T CAL low calibrator CAL L<br>2 vials ADVIA Centaur COV2T CAL high calibrator CAL H<br>ADVIA Centaur COV2T CAL calibrator assigned value sheets and barcode labels	500

Materials Required but Not Provided

The following materials are required to perform these assays, but are not provided:

REF	Description	
	ADVIA Centaur XP system[a]<br>ADVIA Centaur XPT system[a]	
03333963	ADVIA Centaur Probe Wash 3 (probe wash)	50.0 mL PW 3
01137199 (112351)	ADVIA Centaur Wash 1 (wash)	2 x 1500 mL/pack WASH 1
03773025	ADVIA Centaur Wash 1 (wash)	2 x 2500 mL/pack WASH 1

[a] Additional system fluids are required to operate the system: ADVIA Centaur Acid Reagent, ADVIA Centaur Base Reagent, and ADVIA Centaur Cleaning Solution.

Optional Materials

The following materials may be used to perform this assay, but are not provided:

REF	Description	
11206713	ADVIA Centaur COV2T QC (quality control material)	2 x 2.0 mL negative quality control, level 1 CONTROL − 1<br>2 x 2.0 mL positive quality control, level 2 CONTROL + 2<br>Quality control assigned value sheet
07948423 (110314)	ADVIA Centaur Multi-Diluent 2 (diluent)	2 ReadyPack ancillary reagent packs containing 10.0 mL/pack DIL
11207583	ADVIA Centaur COV2T MCM (master curve material)	4 x 1.0 mL levels of master curve material MCM

Assay Procedure

The system automatically performs the following steps:

1. Dispenses 50 µL of sample into a cuvette.
2. Dispenses 100 µL of Solid Phase, then incubates for 3 minutes at 37°C.
3. Dispenses 100 µL of Lite Reagent, then incubates for 6 minutes at 37°C.
4. Performs a wash sequence using ADVIA Centaur Wash 1.
5. Dispenses 300 µL each of ADVIA Centaur Acid Reagent and ADVIA Centaur Base Reagent to initiate the chemiluminescent reaction.
6. Reports results.

Preparing the Reagents

All reagents are liquid and ready to use. Before loading the packs onto the system, reagents require mixing. For information about mixing the reagents, refer to the system online help.

Preparing the System

Ensure that sufficient materials are loaded on the system. Refer to *Materials Provided* and *Materials Required but Not Provided* for guidance about required reagents.

For information about loading products, refer to the system online help.

Master Curve Definition

Before initiating calibration on each new lot of reagent, enter the assay master curve values by scanning the master curve card. For information about defining the master curve, refer to the system online help.

Performing Calibration

For calibration of the ADVIA Centaur COV2T assay, use the calibrators provided with each kit.

Note Calibrators provided in an assay kit must only be used with the reagent lot provided in the same kit.

Calibration Frequency

Perform a calibration if one or more of the following conditions exist:

- At the end of the 14-day calibration interval.
- When changing lot numbers of primary reagent packs.
- When indicated by quality control results.
- After major maintenance or service, if indicated by quality control results.

Follow government regulations or accreditation requirements for calibration frequency. Individual laboratory quality control programs and procedures may require more frequent calibration.

Preparing the Calibrators

Calibrators are liquid and ready to use. Allow the calibrators to equilibrate to room temperature. Gently mix and invert the vials to ensure homogeneity of the material.

Use calibrators within the stability limits specified in *Reagents* and discard any remaining material.

Calibration Procedure

The calibrators are provided in dropper vials. Each dispensed drop is approximately 50 µL.

Perform the calibration procedure using the following steps:

1. Ensure that the appropriate master curve and calibrator assigned values are entered on the system. For information about defining the master curve and entering calibrator values, refer to the system online help.
2. Load the required reagents for the assay.
3. Schedule the calibrators.
4. Label two sample containers with barcode labels: one container for the low calibrator and one container for the high calibrator. Place the barcode labels on the sample containers with the readable characters oriented vertically.

   Note Barcode labels are lot-specific. Do not use barcode labels from one lot of calibrators with any other lot of calibrators.
5. Gently mix the product and dispense a sufficient volume of each calibrator into the appropriate sample containers. Avoid bubbles.

   The required sample volume for testing depends on several factors. For information about sample volume requirements, refer to the system online help.
6. Load the samples according to the system online help.

Note Dispose of any calibrator that remains in the sample container after 8 hours. Do not refill or reuse sample containers. Do not return any calibrator material back into the original container.

Performing Quality Control

For quality control of the ADVIA Centaur COV2T assay, use the ADVIA Centaur COV2T QC or an equivalent product at least once during each day that samples are analyzed. Additional quality control material of known analyte concentration can be used at the discretion of the laboratory. Use the quality control material in accordance with the quality control instructions for use. For the assigned values, refer to the quality control assigned value sheet provided.

A satisfactory level of performance is achieved when the analyte values obtained are within the expected control interval for the system or within your interval, as determined by an appropriate internal laboratory quality control procedure. Follow your laboratory's quality control procedures if the results obtained do not fall within the acceptable limits. For information about entering quality control definitions, refer to the system online help.

Follow government regulations or accreditation requirements for quality control frequency. Individual laboratory quality control programs and procedures may require more frequent quality control testing.

Test quality control samples after a successful calibration.

Taking Corrective Action

If the quality control results do not fall within the expected control interval, do not report results. Perform corrective actions in accordance with established laboratory protocol. For suggested protocol, refer to the system online help.

Results

Calculation of Results

The system determines the result using the calculation procedure described in the system online help. The system reports results in Index Values or U/mL, depending on the units defined when setting up the assay. Refer to *Interpretation of Results*.

Conversion formula: 1.00 Index Value = 1.00 U/mL

For information about results outside the specified measuring interval, refer to *Analytical Measuring Interval*.

Dilutions

The analytical measuring interval is 0.60–45.00 Index (U/mL). For information about dilution options, refer to the system online help.

Dilute and retest samples with values > 45.00 Index (U/mL) to obtain accurate results.

Note Due to the heterogeneity of SARS-CoV-2 antibodies, some patient samples may exhibit a non-linear dilution.

For automated dilutions, perform the following activities.

- Load ADVIA Centaur Multi-Diluent 2.
- Ensure that sufficient sample volume is available. Refer to the table below.
- Select the appropriate dilution factor.

For automatic dilutions, enter a dilution setpoint ≤ 45 Index (U/mL).

For additional instructions on running automatic dilutions, refer to the system online help.

Sample	Dilution	Sample Volume (µL)
Serum and plasma	1:5	40

Interpretation of Results

The system reports ADVIA Centaur COV2T assay results in Index Values or U/mL and as Nonreactive or Reactive:

- **Nonreactive:** < 1.00 Index (U/mL). These samples are considered negative for SARS-CoV-2 antibodies.
- **Reactive:** ≥ 1.00 Index (U/mL). These samples are considered positive for SARS-CoV-2 antibodies.

Results of this assay should always be interpreted in conjunction with the patient's medical history, clinical presentation, and other findings.

Limitations

The following information pertains to limitations of the assay:

- Performance characteristics have not been established for the assay used in conjunction with other manufacturers' assays for specific SARS-CoV-2 serological markers. Laboratories are responsible for establishing their own performance characteristics.
- Results are not intended to be used as the sole basis for patient management decisions. Test results should be interpreted in conjunction with clinical observations, patient history, epidemiological information, and other laboratory findings.
- The performance of the assay has not been established with cord blood, neonatal specimens, cadaver specimens, or body fluids other than serum or plasma.
- It is currently unknown how long SARS-CoV-2 antibodies persist following infection and if the presence of antibodies confers protective immunity.
- A nonreactive test result does not exclude the possibility of exposure to or infection with SARS-CoV-2. Patient specimens may be nonreactive if collected during the early (pre-seroconversion) phase of illness or due to a decline in titer over time. In addition, the immune response may be depressed in elderly, immunocompromised, or immunosuppressed patients.
- Results obtained with the assay may not be used interchangeably with values obtained with different manufacturers' assay methods.
- This test should not be used for donor screening to prevent SARS-CoV-2 transmission during blood, tissue, or organ donations.

Performance Characteristics

The reagent formulations used on the ADVIA Centaur XP and ADVIA Centaur XPT systems are the same as those used on the Atellica® IM Analyzer. Some performance characteristics for the ADVIA Centaur COV2T assay were established using the Atellica IM Analyzer.

Assay performance characteristics are representative data. Results obtained at individual laboratories may vary from the data presented.

Analytical Measuring Interval

0.60–45.00 Index (U/mL) is reported as Nonreactive (< 1.00 Index (U/mL)) or Reactive (≥ 1.00 Index (U/mL)).

The lower limit of the analytical measuring interval is defined by the LoQ (0.60 Index (U/mL)). When sample results exceed the upper limit of the analytical measuring interval, refer to *Dilutions*.

Detection Capability

Detection capability was determined in accordance with CLSI Document EP17-A2.[24] The following results were obtained:

Method	Result<br>Index (U/mL)
Limit of Blank (LoB)	0.30
Limit of Detection (LoD)	0.50
Limit of Quantitation (LoQ)	0.60

Results obtained at individual laboratories may vary from the data presented.

The LoB corresponds to the highest measurement result that is likely to be observed for a blank sample with a probability of 95%. The LoB based on 3 reagent lots is 0.30 Index (U/mL).

The LoD corresponds to the lowest concentration of total antibodies to SARS-CoV-2 that can be detected with a probability of 95%. The LoD based on 3 reagent lots is 0.50 Index (U/mL).

The LoQ corresponds to the lowest concentration of total antibodies to SARS-CoV-2 in a sample at which the within laboratory CV is ≤ 20%. The LoQ of the assay based on 3 reagent lots is 0.60 Index (U/mL).

The lower limit of the analytical measuring interval is defined by the LoQ (0.60 Index (U/mL)).

Seroconversion Sensitivity

A total of 421 specimens were collected serially from 86 subjects with a clinical diagnosis of COVID-19 based on a positive SARS-CoV-2 polymerase chain reaction (PCR) method. Of these, seroconversion was observed in 13 panels with 2 or more nonreactive blood draws and 2 or more reactive blood draws with the ADVIA Centaur COV2T assay using the ADVIA Centaur XP system. The results are shown in the table below:

			First Draw		Last Nonreactive Draw		First Reactive Draw		Last Draw	
Panel	Number of Draws	Number of Reactive Draws	Days Post PCR Positive	Index (U/mL)	Days Post PCR Positive	Index (U/mL)	Days Post PCR Positive	Index (U/mL)	Days Post PCR Positive	Index (U/mL)
A	8	6	5	0.33	6	0.72	7	2.99	12	> 45.00
B	8	6	3	0.18	4	0.51	5	1.26	12	31.33
C	5	2	0	0.13	4	0.57	8	> 45.00	10	> 45.00
D	8	6	3	0.24	4	0.60	5	1.47	32	26.35
E	5	3	0	0.28	2	0.92	3	4.01	5	42.61
F	8	6	0	0.27	3	0.56	4	1.18	12	41.59
G	6	4	5	0.27	6	0.59	7	1.72	10	20.34
H	7	5	0	0.27	2	0.68	3	2.44	7	> 45.00
I	5	2	0	0.26	3	0.24	14	26.84	17	31.51
J	4	2	0	0.20	6	0.21	13	11.14	17	10.49
K	4	2	2	0.22	4	0.87	6	16.15	22	> 45.00
L	8	4	0	0.24	12	0.37	16	13.87	26	24.38
M	5	2	0	0.28	5	0.93	10	> 45.00	12	> 45.00

Clinical Sensitivity and Specificity

Clinical sensitivity and specificity were determined in accordance with CLSI Document EP12-A2.[25] The performance of the ADVIA Centaur COV2T assay was determined by testing a total of 2162 samples using the ADVIA Centaur XP system.

Results obtained at individual laboratories may vary from the data presented.

Clinical Sensitivity

Clinical sensitivity was determined by testing 573 samples collected from 238 unique donor subjects with a clinical diagnosis of COVID-19 based on a positive polymerase chain reaction (PCR) method. The following table describes clinical sensitivity by time of sampling following a positive PCR result:

Days Post PCR Positive	Number Tested	Reactive	Nonreactive	Clinical Sensitivity (95% CI)
0–6	268	164	104	61.19% (55.08%–67.06%)
7–13	174	160	14	91.95% (86.87% –95.53%)
14–20	53	52	1	98.11% (89.93% –99.95%)
≥ 21	78	78	0	100% (95.38% –100%)

Clinical sensitivity from the date of symptom onset was determined by analyzing a subset of the 573 samples (372 samples collected from 203 unique donor subjects). The results are shown in the table below:

Days Post Symptom Onset	Number Tested	Reactive	Nonreactive	Clinical Sensitivity (95% CI)
0–6	81	39	42	48.15% (37.60%–58.86%)
7–13	126	99	27	78.57% (70.62%–84.83%)
14–20	61	55	6	90.16% (80.16%–95.41%)
≥ 21	104	104	0	100% (96.44%–100%)

Clinical Specificity

Clinical specificity was determined by testing 1589 samples collected prior to the COVID-19 outbreak (before November 2019) from apparently healthy individuals and apparently healthy pregnant women in the United States. The results are shown in the table below:

Group	Number Tested	Nonreactive	Reactive	Clinical Specificity (95% CI)
Apparently Healthy	1489	1486	3	99.80% (99.41%–99.96%)
Apparently Healthy Pregnant Women	100	100	0	100% (96.38%–100%)
**Total**	**1589**	**1586**	**3**	**99.81% (99.45%–99.96%)**

Correlation to Plaque Reduction Neutralization Test (PRNT)

The correlation of 50% PRNT neutralization titer ($PRNT_{50}$) to Index values (U/mL) was evaluated by testing 29 samples from subjects with a clinical diagnosis of COVID-19 based on a positive SARS-CoV-2 PCR method and above the 1:20 $PRNT_{50}$ cutoff.

All samples were reactive with the Atellica IM COV2T assay (Sensitivity = 100% [29/29], 95% CI: 88.06%–100%).

There were 19 samples within the Atellica IM COV2T analytical measuring interval. A quantitative correlation of Atellica IM COV2T Index (U/mL) versus $PRNT_{50}$ was determined by linear regression and Pearson correlation. The correlation coefficient is 0.795, demonstrating a strong relationship between the Atellica IM COV2T assay Index values (U/mL) and $PRNT_{50}$, as shown in the graph below.

Atellica IM COV2T Assay Comparison to 50% PRNT

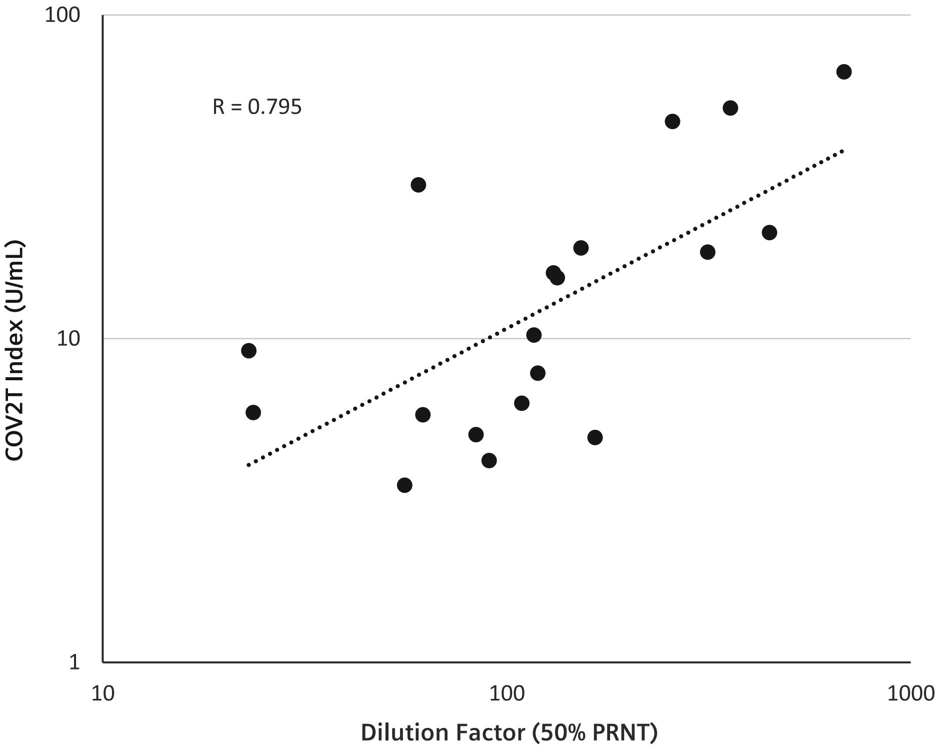

SARS-CoV-2 Variant Detection

Multiple SARS-CoV-2 variants emerged in late 2020 and are circulating globally. One variant of concern, the P.1 variant, has 17 unique mutations, including 3 amino acid substitutions (N501Y, E484K, and K417T) in the receptor binding domain of the spike protein. Serum samples from 14 confirmed P.1 variant infected individuals were collected 19–30 days following a COVID-19 diagnosis by RT-PCR. All samples were reactive with the ADVIA Centaur COV2T assay using the ADVIA Centaur XP system, with results ranging from 2.71 Index (U/mL) to > 45.00 Index (U/mL).

Precision

Precision was determined in accordance with CLSI Document EP05-A3.[26] Samples were assayed in duplicate in 2 runs per day for 20 days using the ADVIA Centaur XP system.

Specimen Type	N[a]	Mean Index (U/mL)	Repeatability		Within-Laboratory Precision	
			SD[b] Index (U/mL)	CV[c] (%)	SD Index (U/mL)	CV (%)
Plasma A	80	3.36	0.074	2.2	0.163	4.8
Plasma B	80	6.38	0.119	1.9	0.346	5.4
Plasma C	80	20.73	0.451	2.2	1.207	5.8
Plasma D	80	44.43	1.121	2.5	3.541	8.0
Control 1	80	0.08	0.039	N/A[d]	0.060	N/A
Control 2	80	2.16	0.044	2.0	0.084	3.9

[a] Number of measurements.
[b] Standard deviation.
[c] Coefficient of variation.
[d] Not applicable.

The assay was designed to have the following precision.

Concentration Interval	Precision	
Index (U/mL)	Repeatability (Within-Run)	Within-Laboratory (Total Precision)
0.70–2.00	≤ 12.0% CV	≤ 15.0% CV
> 2.00	≤ 10.0% CV	≤ 12.0% CV

Results obtained at individual laboratories may vary from the data presented.

Specimen Equivalency

Specimen equivalency was determined with the Deming regression model using the ADVIA Centaur XP system in accordance with CLSI Document EP35-Ed1.[27]

Results from plasma draws were compared to serum draws. The following results were obtained:

Tube (y) vs. Serum (x)	N[a]	Sample Interval	Slope	Intercept	r[b]
EDTA (plasma)	39	0.54–38.02 Index (U/mL)	0.92	0.15	0.996
Lithium heparin (plasma)	39	0.99–42.94 Index (U/mL)	1.03	-0.02	0.994

a Number of samples tested.
b Correlation coefficient.

Results from capillary draws were compared to venipuncture draws. The following results were obtained:

Capillary Puncture (y) vs. Venipuncture (x)	N[a]	Sample Interval	Slope	Intercept	r[b]
Capillary draw	23	0.38–44.74 Index (U/mL)	1.03	-0.82	0.987

a Number of samples tested.
b Correlation coefficient.

The assay is designed to have a slope of 0.90–1.10 for alternate sample types.

Agreement of the specimen types may vary depending on the study design and sample population used. Results obtained at individual laboratories may vary from the data presented.

Interferences

Interference testing was performed in accordance with CLSI Document EP07-ed3[28] and EP37-ed1[29]. Results were established with the Atellica IM COV2T assay using the Atellica IM Analyzer. Testing demonstrated a ≤ 10% change for each substance at the indicated concentration.

Substance	Substance Test Concentration
Hemoglobin	1000 mg/dL
Bilirubin, conjugated	40 mg/dL
Bilirubin, unconjugated	40 mg/dL
Triglycerides (Intralipid)	2000 mg/dL
Biotin	3500 ng/mL
Protein, total	15 g/dL
Cholesterol	500 mg/dL

Cross-Reactivity

Cross-reactivity was determined in accordance with CLSI Document EP07-ed3.[28] The assay was evaluated for potential cross-reactivity using specimens containing antibodies to other pathogens and other disease states using the ADVIA Centaur COV2T assay with the ADVIA Centaur XP system. The results are shown in the table below:

Clinical Category	Number Tested	Number Reactive with ADVIA Centaur COV2T Assay
Autoimmune diseases[a]	15	0
Candida albicans antibody	10	1

Clinical Category	Number Tested	Number Reactive with ADVIA Centaur COV2T Assay
Chlamydia pneumoniae IgG	10	0
Chlamydia trachomatis IgM	5	0
Cytomegalovirus (CMV) IgG	15	0
Cytomegalovirus (CMV) IgM	5	0
Epstein Barr virus (EBV) IgG	5	0
Epstein Barr virus (EBV) IgM	5	0
Haemophilus influenzae b (Hib) IgG	20	0
Hepatitis A virus (HAV) IgM	5	0
Hepatitis B core (anti-HBc) IgM	5	0
Hepatitis C virus (HCV) antibody	5	0
Herpes simplex virus (HSV) IgM	3	0
Human anti-mouse antibody (HAMA)	5	0
Human coronavirus antibodies[b]	29	0
Human immunodeficiency virus (HIV) antibody	10	0
Human metapneumovirus (HMPV) IgG	5	0
Influenza antibody	29	0
Influenza A antibody	6	0
Influenza B antibody	10	0
Measles antibody	5	0
Middle East respiratory syndrome coronavirus (MERS-CoV) IgG	5	0
Mycoplasma pneumoniae IgG	19	0
Parvovirus B19 antibody	5	0
Respiratory pathogen antibodies[c]	23	0
Respiratory syncytial virus (RSV) antibody	20	0
Severe acute respiratory syndrome coronavirus (SARS-CoV-1) IgG	5	0
Streptococcus pneumoniae anti-PCP IgG	10	0
Toxoplasma gondii antibody	10	0
Toxoplasma gondii IgG	20	1

Clinical Category	Number Tested	Number Reactive with ADVIA Centaur COV2T Assay
Varicella zoster virus (VZV) antibody	5	0
**Total**	**329**	**2**

[a] This group consists of samples from 14 subjects with autoimmune disease states, including anti-nuclear antibody (ANA; N = 5), Graves' disease (N = 5), and rheumatoid factor (RF; N = 5).

[b] This panel includes 29 subjects who had antibodies to multiple human coronaviruses, including coronavirus HKU (N = 24), coronavirus OC43 (N = 27), coronavirus 229E (N = 29), coronavirus NL63 (N = 21).

[c] This panel consists of samples from 23 subjects with antibodies to multiple respiratory pathogens, including Adenovirus antibodies (N = 8), *Bordetella pertussis* IgG (N = 19), *Chlamydia pneumoniae* IgG (N =23), *Chlamydia psittaci* IgG (N = 3), *Chlamydia psittaci* IgM (N = 1), *Haemophilus influenzae* b (Hib) IgG (N = 11), Influenza A IgG (N = 22), Influenza A IgM (N = 1), Influenza B IgG (N = 18) , Influenza B IgM (N = 1), and *Mycoplasma pneumoniae* IgG (N = 6).

Results obtained at individual laboratories may vary from the data presented.

Linearity

Linearity testing was performed in accordance with CLSI Document EP06-A.[30]

Patient pools containing high levels of SARS-CoV-2 total antibodies (1 serum, 1 EDTA plasma, and 1 lithium heparin plasma) were diluted with the respective negative pool to prepare a dilution series comprised of a minimum of ten (10) levels. Each level was tested in 3 replicates using the ADVIA Centaur XP system.

Linearity was demonstrated for the analytical measuring interval of 0.60–45.00 Index (U/mL) using the ADVIA Centaur XP system with deviations from linearity within 15%.

Onboard Dilution Recovery

Samples including serum, lithium heparin plasma, and EDTA plasma in the range of 47.46–111.37 Index (U/mL) were diluted 1:5 with ADVIA Centaur Multi-Diluent 2 and assayed for recovery using the ADVIA Centaur XP system. The recoveries ranged from 87.3%–115.7%. **Note** Onboard dilution recovery was not assessed in a population vaccinated against COVID-19.

The extended measuring interval of the ADVIA Centaur COV2T assay by dilution of 1:5 with ADVIA Centaur Multi-Diluent 2 is 45.00–225 Index (U/mL).

Sample	Dilution	Observed Index (U/mL)	Expected Index (U/mL)	Recovery (%)
Serum 1	—	94.67	—	—
	1:5	21.90	18.93	115.7
Serum 2	—	96.61	—	—
	1:5	17.42	19.32	90.2
Lithium heparin plasma 1	—	86.61	—	—
	1:5	16.55	17.32	95.5
Lithium heparin plasma 2	—	74.48	—	—
	1:5	15.50	14.90	104.0
EDTA Plasma 1	—	78.43	—	—
	1:5	13.70	15.69	87.3

Sample	Dilution	Observed Index (U/mL)	Expected Index (U/mL)	Recovery (%)
EDTA Plasma 2	—	111.37	—	—
	1:5	21.47	22.27	96.4
EDTA Plasma 3	—	47.46	—	—
	1:5	10.66	9.49	112.3
**Mean**				**100.2**

Standardization

The ADVIA Centaur COV2T assay standardization is traceable to an internal standard based on agreement with known positive and negative SARS-CoV-2 samples. The internal standardization supports reporting of results in Index Values or U/mL.

The analytical sensitivity at the cut-off values for the ADVIA Centaur COV2T assay was determined on the ADVIA Centaur XP system using the World Health Organization (WHO) 1st International Standard for anti-SARS-CoV-2 immunoglobulin (human) NIBSC code: 20/136. The concentration of the reference standard that corresponds to the cut-off value of 1.00 Index (U/mL) for the ADVIA Centaur COV2T assay is 6.57 BAU/mL.

Technical Assistance

For customer support, contact your local technical support provider or distributor.

siemens-healthineers.com

References

1. Lai CC, Shih TP, Ko WC, Tang HJ, Hsueh PR. Severe acute respiratory syndrome coronavirus 2 (SARS-CoV-2) and coronavirus disease-2019 (COVID-19): The epidemic and the challenges. *Int J Antimicrob Agents*. 2020;55(3):105924.
2. Guo YR, Cao QD, Hong ZS, Tan YY, Chen SD, Jin HJ, *et al*. The origin, transmission and clinical therapies on coronavirus disease 2019 (COVID-19) outbreak - an update on the status. *Mil Med Res*. 2020;7(1):11.
3. Li H, Liu SM, Yu XH, Tang SL, Tang CK. Coronavirus disease 2019 (COVID-19): current status and future perspectives. *Int J Antimicrob Agents*. 2020;29:105951.
4. Wu Z and McGoogan JM. Characteristics of and Important Lessons From the Coronavirus Disease 2019 (COVID-19) Outbreak in China: Summary of a Report of 72314 Cases From the Chinese Center for Disease Control and Prevention. *JAMA* 2020;323(13):2648.
5. Pan A, Liu L, Wang C, Guo H, Hao X, Wang Q, *et al*. Association of Public Health Interventions With the Epidemiology of the COVID-19 Outbreak in Wuhan, China. *JAMA*. 2020:6130.
6. Rothan HA and Byrareddy SN. The epidemiology and pathogenesis of coronavirus disease (COVID-19) outbreak. *J Autoimmun*. 2020;109:102433.
7. Lai CC, Liu YH, Wang CY, Wang YH, Hsueh SC, Yen MY, *et al*. Asymptomatic carrier state, acute respiratory disease, and pneumonia due to severe acute respiratory syndrome coronavirus 2 (SARS-CoV-2): Facts and myths. *J Microbiol Immunol Infect*. 2020;pii: S1684-1182;(20):30040-2.
8. Wu C, Chen X, Cai Y, Xia J, Zhou X, Xu S, *et al*. Risk Factors Associated With Acute Respiratory Distress Syndrome and Death in Patients With Coronavirus Disease 2019 Pneumonia in Wuhan, China. *JAMA Intern Med*. 2020;10:1001.

9. Rodriguez-Morales AJ, Cardona-Ospina JA, Gutiérrez-Ocampo E, Villamizar-Peña R, Holguin-Rivera Y, Escalera-Antezana JP, *et al*. Clinical, laboratory and imaging features of COVID-19: A systematic review and meta-analysis. *Travel Med Infect Dis*. 2020;13:101623.
10. Centers for Disease Control and Prevention (CDC). Coronoavirus Disease 2019: Evaluation and Testing page. https://www.cdc.gov/coronavirus/2019-nCoV/hcp/clinical-criteria.html. Accessed April 27, 2020
11. World Health Organization. Coronavirus disease (COVID-19) technical guidance: Laboratory testing for 2019-nCoV in humans page. https://www.who.int/emergencies/diseases/novel-coronavirus-2019/technical-guidance/laboratory-guidance. Accessed April 27, 2020
12. Jin YH, Cai L, Cheng ZS, Cheng H, Deng T, Fan YP, *et al*. A rapid advice guideline for the diagnosis and treatment of 2019 novel coronavirus (COVID-19) infected pneumonia (standard version). *Mil Med Res*. 2020;7(1):4.
13. Zhao J, Yuan Q, Wang H, Liu W, Liao X, Su Y, *et al*. Antibody responses to SARS-CoV-2 in patients of novel coronavirus disease 2019. *Clin Infect Dis*. 2020;pii:344.
14. Li Z, Yi Y, Luo X, Xiong N, Liu Y, Li S, *et al*. Development and clinical application of a rapid IgM-IgG combined antibody test for SARS-CoV-2 infection diagnosis. *J Med Virol*. 2020;10.1002:25727.
15. Yang JR, Deng DT, Wu N, Yang B, Li HJ, and Pan XB. Persistent viral RNA positivity during recovery period of a patient with SARS-CoV-2 infection. *J Med Virol*. 2020:10.10002:25940.
16. Wu F, Wang A, Liu M, Wang Q, Chen J, Xia S, *et al*. Neutralizing antibody responses to SARS-CoV-2 in a COVID-19 recovered patient cohort and their implications. medRxiv; https://doi.org/10.1101/2020.03.30.20047365. Accessed April 30, 2020.
17. Widge AT, Rouphael NG, Jackson LA, *et al*; mRNA-1273 Study Group. Durability of responses after SARS-CoV-2 mRNA-1273 vaccination. *N Engl J Med*. 2021;384(1):80-82.
18. Centers for Disease Control. Perspectives in disease prevention and health promotion update: Universal precautions for prevention of transmission of human immunodeficiency virus, hepatitis B virus and other bloodborne pathogens in healthcare settings. *MMWR*. 1988;37(24):377–382, 387–388.
19. Clinical and Laboratory Standards Institute. *Procedures for the Handling and Processing of Blood Specimens for Common Laboratory Tests; Approved Guideline—Fourth Edition*. Wayne, PA: Clinical and Laboratory Standards Institute; 2010. CLSI Document GP44-A4.
20. Clinical and Laboratory Standards Institute. *Protection of Laboratory Workers From Occupationally Acquired Infections; Approved Guideline—Fourth Edition*. Wayne, PA: Clinical and Laboratory Standards Institute; 2014. CLSI Document M29-A4.
21. Clinical and Laboratory Standards Institute. *Procedures for the Collection of Diagnostic Blood Specimens by Venipuncture; Approved Standard—Sixth Edition*. Wayne, PA: Clinical and Laboratory Standards Institute; 2007. CLSI Document GP41-A6.
22. Becton, Dickinson and Company. BD Microtainer® Tubes directions for use. 3/2011. Document 8363932.
23. Clinical and Laboratory Standards Institute. *Tubes and Additives for Venous and Capillary Blood Specimen Collection; Approved Standard—Sixth Edition*. Wayne, PA: Clinical and Laboratory Standards Institute; 2010. CLSI Document GP39-A6.
24. Clinical and Laboratory Standards Institute. *Evaluation of Detection Capability for Clinical Laboratory Measurement Procedures; Approved Guideline—Second Edition*. Wayne, PA: Clinical and Laboratory Standards Institute; 2012. CLSI Document EP17-A2.
25. Clinical and Laboratory Standards Institute. *User Protocol for Evaluation of Qualitative Test Performance; Approved Guideline—Second Edition*. Wayne, PA: Clinical and Laboratory Standards Institute; 2008. CLSI Document EP12-A2.

26. Clinical and Laboratory Standards Institute. *Evaluation of Precision of Quantitative Measurement Procedures; Approved Guideline—Third Edition*. Wayne, PA: Clinical and Laboratory Standards Institute; 2014. CLSI Document EP05-A3.
27. Clinical and Laboratory Standards Institute. *Assessment of Equivalence or Suitability of Specimen Types for Medical Laboratory Measurement Procedures—1st Edition*. Wayne, PA: Clinical and Laboratory Standards Institute; 2019. CLSI Document EP35-Ed1.
28. Clinical and Laboratory Standards Institute. *Interference Testing in Clinical Chemistry; Approved Guideline—Third Edition*. Wayne, PA: Clinical and Laboratory Standards Institute; 2018. CLSI Document EP07-ed3.
29. Clinical and Laboratory Standards Institute. *Supplemental Tables for Interference Testing in Clinical Chemistry; Approved Supplement—First Edition*. Wayne, PA: Clinical and Laboratory Standards Institute; 2018. CLSI Document EP37-ed1.
30. Clinical and Laboratory Standards Institute. *Evaluation of the Linearity of Quantitative Measurement Procedures: A Statistical Approach; Approved Guideline*. Wayne, PA: Clinical and Laboratory Standards Institute; 2003. CLSI Document EP06-A.

Definition of Symbols

The following symbols may appear on the product labeling:

Symbol	Symbol Title and Description
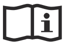	Consult instructions for use
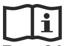 Rev. 01	Version of instructions for use
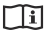 siemens.com/healthcare<br>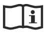 siemens.com/document-library	Internet URL address to access the electronic instructions for use
Rev. REVISION	Revision
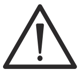	Caution<br>Consult instructions for use or accompanying documents for cautionary information such as warnings and precautions that cannot, for a variety of reasons, be presented on the medical device.
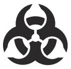	Biological risks<br>Potential biological risks are associated with the medical device.
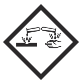	Corrosive
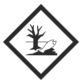	Dangerous to environment
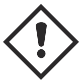	Irritant<br>Oral, dermal, or inhalation hazard
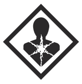	Inhalation hazard<br>Respiratory or internal health

Symbol	Symbol Title and Description
	Flammable<br>Flammable to extremely flammable
	Oxidizing
	Explosive
	Toxic
	Compressed gas
	Keep away from sunlight<br>Prevent exposure to sunlight and heat.
	Up<br>Store in an upright position.
	Do not freeze
2°C 8°C	Temperature limit<br>Upper and lower limits of temperature indicators are adjacent to the upper and lower horizontal lines.
	Handheld barcode scanner
IVD	*In vitro* diagnostic medical device
Σ (n)	Contains sufficient for <n> tests<br>Total number of IVD tests the system can perform with the IVD kit reagents appears adjacent to the symbol.
**RxOnly**	Prescription device (US only)<br>Applies only to United States-registered IVD assays.<br>CAUTION: Federal (USA) law restricts this device to sale by or on the order of a licensed healthcare professional.
	Mixing of substances<br>Mix product before use.
g mL	Reconstitute and mix lyophilized product before use.
	Target
	Interval

Symbol	Symbol Title and Description
	Legal Manufacturer
EC REP	Authorized Representative in the European Community
	Use-by date<br>Use by the designated date.
LOT	Batch code
REF	Catalog number
	Recycle
PRINTED WITH SOY INK	Printed with soy ink
CE	CE Mark
CE XXXX	CE Mark with notified body ID number<br>Notified body ID number can vary.
YYYY-MM-DD	Date format (year-month-day)
CHECKSUM	Variable hexadecimal number that ensures the Master Curve and Calibrator definition values entered are valid.
MC DEF	Master Curve Definition
LOT DTL	Lot Details
UNITS C	Common Units
UNITS SI	International System of Units
MATERIAL	Material
MATERIAL ID	Unique material identification number
CONTROL NAME	Name of control
CONTROL TYPE	Type of control

Legal Information

Atellica, ReadyPack, and ADVIA Centaur are trademarks of Siemens Healthcare Diagnostics.

All other trademarks and brands are the property of their respective owners.

© 2020-2021 Siemens Healthcare Diagnostics. All rights reserved.

Siemens Healthcare Diagnostics Inc.
511 Benedict Avenue
Tarrytown, NY 10591
USA
siemens-healthineers.com

Global Siemens Headquarters
Siemens AG
Wittelsbacherplatz 2
80333 Muenchen
Germany

Siemens Healthcare Headquarters
Siemens Healthcare GmbH
Henkestr. 127
91052 Erlangen
Germany
Phone: +49 9131 84-0
siemens-healthineers.com

Global Division
Siemens Healthcare Diagnostics Inc.
511 Benedict Avenue
Tarrytown, NY 10591
USA
siemens-healthineers.com
